# Many quality measurements, but few quality measures assessing the quality of breast cancer care in women: A systematic review

**DOI:** 10.1186/1471-2407-6-291

**Published:** 2006-12-18

**Authors:** Howard M Schachter, Vasil Mamaladze, Gabriela Lewin, Ian D Graham, Melissa Brouwers, Margaret Sampson, Andra Morrison, Li Zhang, Peter O'Blenis, Chantelle Garritty

**Affiliations:** 1 Children's Hospital of Eastern Ontario Research Institute, 401 Smyth Road, Ottawa, ON K1H 8L1, USA; 2 Ottawa Health Research Institute, 1053 Carling Ave, ASB Room 2-008, Ottawa, ON K1Y 4E9, USA; 3 McMaster University, 1280 Main St West, Rm 314, Hamilton, ON L8S 4L8, USA; 4 Canadian Coordinating Office for Health Technology Assessment, 600-865 Carling Ave, Ottawa, ON K1S 5S8, USA; 5 Health Sciences Library, Rm B205, University of Saskatchewan, 107 Wiggins Rd, Saskatoon, SK S7N 5E5, USA; 6 TrialStat Corporation, 44 ByWard Market Square, Suite 270, Ottawa, ON K1N 7A2, USA

## Abstract

**Background:**

Breast cancer in women is increasingly frequent, and care is complex, onerous and expensive, all of which lend urgency to improvements in care. Quality measurement is essential to monitor effectiveness and to guide improvements in healthcare.

**Methods:**

Ten databases, including Medline, were searched electronically to identify measures assessing the quality of breast cancer care in women (diagnosis, treatment, followup, documentation of care). Eligible studies measured adherence to standards of breast cancer care in women diagnosed with, or in treatment for, any histological type of adenocarcinoma of the breast. Reference lists of studies, review articles, web sites, and files of experts were searched manually. Evidence appraisal entailed dual independent assessments of data (e.g., indicators used in quality measurement). The extent of each quality indicator's scientific validation as a measure was assessed. The American Society of Clinical Oncology (ASCO) was asked to contribute quality measures under development.

**Results:**

Sixty relevant reports identified 58 studies with 143 indicators assessing adherence to quality breast cancer care. A paucity of validated indicators (n = 12), most of which assessed quality of life, only permitted a qualitative data synthesis. Most quality indicators evaluated processes of care.

**Conclusion:**

While some studies revealed patterns of under-use of care, all adherence data require confirmation using validated quality measures. ASCO's current development of a set of quality measures relating to breast cancer care may hold the key to conducting definitive studies.

## Background

Cancer is the second-most common cause of death (after cardio-vascular disease) in North Americans, and breast cancer is the most commonly diagnosed cancer in women [1]. It was estimated in 2004 that one in seven American women would develop breast cancer in her lifetime [1], up from one in eight estimated in 2003 [2].

During the 1990's, American women's 5-year breast cancer survival rates improved on average 2.3% per year, with the largest improvements for younger women. However, survival rates are generally lower for African American women, with 30% excess deaths compared with white women estimated in the year 2000 [3]. Thus, while some aspects of breast cancer care (e.g., earlier detection) have contributed to improved survival, increasing rates of disease and disparities in outcomes point to outstanding issues to be addressed.

The enormous toll on women, families and society make it urgent that breast cancer care be as effective, safe, accessible and equitable as possible. The foundation of this investigation must be sound research to refine what represents "quality care" (e.g., timely access to efficacious and safe treatments). Only by measuring and monitoring adherence to recommended care can meaningful trends and gaps in the delivery, receipt and outcomes of care be identified and put in context, at all levels, from individual centers to nationally and globally [4].

Health care quality measurement is an emerging field, developing alongside the establishment of goals for health care delivery and utilization. Ideally, stakeholders within the health care system will assess internal quality improvement and accountability, and oversee external health care quality, by appropriate measurement of the rates of adherence to recommended care. This would guide policy, the provision of care, and future research directions.

The quality of health care is "the degree to which healthcare services ... increase the likelihood of desired health outcomes and are consistent with current professional knowledge" [5]. Despite more than a trillion dollars spent annually on health care in general in the USA, however, it is suggested that the care received by Americans falls well short of ideal [6]. On average, almost half of those in need do not receive recommended care [7,8].

Health care quality measurement may address a question such as: How many women in a given clinical situation (e.g., diagnosis, treatment history) receive a standard of care (e.g., radiation following surgery) within a specific time frame? Similar questions, yet ones which might yield different results, could be: How many health care practitioners offer or deliver a particular standard of care to women in a specific clinical situation? Patient refusal of care may account for discrepancies in rates identified by these questions.

The assessment of the delivery or receipt of quality health care may seem deceptively straightforward, with large quantities of data available in health care records or cancer registries, for example. These data sources permit the measurement of rates of adherence to recommended health care *processes *(e.g., a competent and timely action by the health care practitioner), *structures *(e.g., the availability of diagnostic imaging equipment), or *outcomes *(e.g., event-free survival; quality of life).

It is not sufficient simply to compile information from health records, health care providers or patients related to a definition of quality care (e.g., if diagnosis X, then deliver care Y within Z weeks). Scientific validation is needed to ensure that data specifically and repeatedly reflect details defining the care in question; that measurements accurately reflect patterns of practice. Indeed, without ensuring scientific soundness, a definition given to individuals extracting data from medical records, or used to solicit information from other data sources (e.g., patients), may complicate or even prevent the identification of what was intended. Unless health care indicators survive the rigors of a scientific process and are found to have sound psychometric properties, they cannot formally be considered quality "measures" *per se*. In the absence of validated measures, observations may be misleading.

What, then, are the requirements for the development of a sound quality measure?

The definition of quality care should be evidence-based [7], possibly with a subsequent expert consensus process, and with details that are precisely expressed (e.g., in a clinical practice guideline). For example, quality care for women with early stage breast cancer entails the receipt of radiotherapy following breast-conserving surgery. Evidence from randomized controlled trials (RCTs) has shown that this less invasive, less disfiguring strategy brings survival outcomes identical to those following mastectomy. This definition of quality care is considered an indicator of quality care, or quality indicator (e.g., if early stage breast cancer in women, then radiotherapy following breast-conserving surgery within a specific time-frame). Establishing the rate of adherence to this quality (care) indicator according to a specific data source amounts to quality measurement.

The definition of a quality indicator must be specific, complete, and clearly worded regarding, for instance, the target population (e.g., women with specific diagnoses) and the characteristics of the care (e.g., the order, type and timing of care). It must be verified that different users share the same meaning and therefore make the same observations when, on different occasions, they consult various data sources (e.g., clinic or hospital records) to gather data. This verifies an indicator's *reliability *as a quality measure.

Additional scientific validation is necessary to increase the confidence that the measured rate of adherence reflects the actual delivery/receipt of particular care (e.g., "percentage of women receiving radiotherapy after breast-conserving surgery"). Along with reliability, sound *validity *indicates and ensures that observations unambiguously reflect what was intended to be identified. For example, only data pertaining to the details circumscribed by the quality indicator should be sought and collected (e.g., the request to identify clinical outcome data should not result in extraction of data for surrogate measures).

### Project Scope

Our rationale in conducting this systematic review was to identify extant quality measures, which could be employed by stakeholders (e.g., service providers) to assure or improve the quality of breast cancer care in women. While it is our view that validated quality measures are required to appropriately ascertain the quality of breast cancer care in women, both formally developed quality measures, as well as quality indicators having received little or no scientific development, were eligible for inclusion in our review. It was thought that it would add value to this project, but practical constraints made it impossible to evaluate the soundness of the empirical evidence supporting recommended standards for care.

## Methods

A seven-member Technical Expert Panel (TEP) provided advisory support, including refining the questions, highlighting key variables requiring consideration in the evidence synthesis and supporting refinement of the scope of the project. Detailed methods information, including the search strategy and data assessment/abstraction forms is available elsewhere [9].

### Study Identification

Various electronic bibliographic databases (Medline, Cancerlit, Healthstar, Premedline, Embase, CINAHL, Cochrane Database of Systematic Reviews, Database of Abstracts of Reviews of Effectiveness, Cochrane Central Register of Controlled Trials, and, Health and Psychosocial Instruments (HAPI)) were searched for reports published from 1992 to 2003 relevant to breast cancer diagnosis and treatment, and quality measures. Another search to retrieve systematic reviews of breast cancer treatment or diagnosis was executed in Medline and Cancerlit, with retrieval limited to material published after 1993. Additional published or unpublished literature was sought through manual searches of reference lists of included studies and key review articles, and from the files of content experts. Web sites were searched, including AHRQ's National Quality Measures Clearinghouse. The American Society of Clinical Oncologists (ASCO) was at that time developing care quality measures, but wished first to complete its work before disseminating it. After removing duplicate citations via Reference Manager™ (Thomson ResearchSoft, Carlsbad, CA.), bibliographic records were identified and posted to a secure internet-based software system for review.

Following calibration exercises, bibliographic records (level 1), and then retrieved articles (level 2) were screened for relevance, with two reviewers per stage. A final screening (level 3) excluded reports describing clinical practice guidelines, systematic reviews, and commentaries/editorials that had initially passed into data abstraction before the project scope was narrowed to exclude examination of the strength of the empirical evidence supporting any given recommended breast cancer care. Disagreements were resolved by consensus and, if necessary, third party intervention. Excluded studies were noted as to the reason for their ineligibility using a modified QUOROM format [10].

### Inclusion/Exclusion Criteria

The population of interest was female adults, diagnosed with or in treatment for breast cancer, including all histological types of adenocarcinoma, both *in situ *and invasive. Quality measurement efforts had to have focused on at least one data source (e.g., medical records; cancer registries; patient or provider questionnaires), entailed any sampling strategy (e.g., convenience sample over a period of time in a health care setting; hospital medical records; general population sample from a given region) and could index any domain (e.g., structure; process).

Searches were restricted to post-1992 because, in the opinion of the funders, quality measurement efforts concerning breast cancer care began to receive serious attention in the ten years prior to the initiation of this project.

Quality indicators could be derived from any source (e.g., clinical practice guideline) and have been subjected to any degree of scientific development, but reference had to have been made to the empirical evidence supporting each indicator. A standard of care (e.g., a recommendation in a guideline) serving as the basis for quality measurement had to have been established prior to the quality measurement effort, so that it would have been available at the time to guide the care subsequently assessed using the quality indicator. Given the unique issues related to breast cancer, measures of quality of life (QOL) and patient satisfaction had to have been developed or adapted for use with breast cancer patients. Inflammatory breast cancer, Paget's disease, phyllodes tumors, and benign breast conditions were excluded. A separate initiative is addressing breast cancer screening and prevention.

### Data Abstraction

Following a calibration exercise involving two studies, three reviewers independently abstracted the contents of each included study using an electronic data abstraction form. Abstracted data were then verified by a second reviewer. Data included: report characteristics (e.g., publication status); study characteristics (e.g., data sources); population characteristics (e.g., case characteristics [size of tumor; level of lymph node involvement; presence/absence of metastasis]); characteristics of the quality indicators used in quality assessment (e.g., data concerning reliability, validity, and study-obtained links to outcomes; whether data extractors were trained and extractions were independently verified) [10]; and adherence data (e.g., overall adherence rate; variations in rates based on review-relevant stratifications such as age; possible reasons for failure to receive care, including patient refusal).

After a calibration exercise involving two included studies, each quality indicator was assessed independently by two reviewers to determine the extent of its scientific development as a quality measure. Levels of development were:

I – quality indicator was developed prior to its implementation in the present study, according to scientific principles (e.g., assessment of scientific soundness, feasibility and ease of use, reliability, internal validity, sensitivity, and pilot testing with appropriate rigor and relevant data sources);

II – quality indicator was being actively developed as part of the present quality measurement study;

III – quality indicator was not currently under development, but existing psychometric data were reported; or,

IV – quality indicator was not currently under development and no psychometric data were reported. Levels I-III could be further subdivided according to the soundness of the reported psychometric properties.

### Data Synthesis

Data from relevant studies were synthesized qualitatively, including: diagnosis; treatment (including supportive care); followup care; and the reporting/documentation of care.

Variables to be taken into consideration included the study population (e.g., age, race/ethnicity, socioeconomic status), data sources (e.g., cancer registries), sampling techniques (e.g., convenience sample, random general population sample), and the purpose of the indicators/measures (e.g., internal quality improvement). Other parameters of interest included measurements of outcomes linked to the quality measurements, and psychometric properties of the identified quality measures (e.g., sensitivity and specificity for diagnostic tests).

Quantitative syntheses of adherence data were not possible, given the paucity of data from validated measures.

## Results

Lists of included and excluded studies (with reasons for exclusion), evidence and summary tables and a comprehensive report are available electronically elsewhere [9]

**Figure 1:**
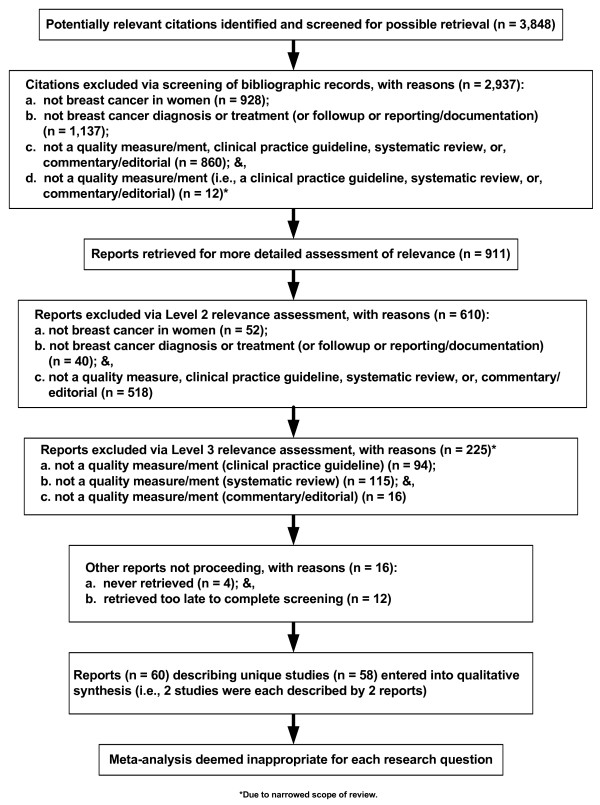
Modified QUOROM Flow Chart.

Results of record retrieval and screening are summarized in Figure 1. From 3,848 unique records identified at the outset, 60 reports, describing 58 studies, met eligibility criteria, and 143 quality indicators were identified (Table 1).

Many different populations were investigated, typically retrospectively, using various reference standards (e.g., clinical practice guidelines) and data sources (e.g., medical records). Younger women, and those with early stage breast cancer, were more likely to have been studied. Most standards reflected processes of care, focusing most often on whether or not women with breast cancer received indicated care (e.g., percentage of women treated with breast-conserving surgery who begin radiation therapy within 6 weeks of completing either of the following: the last surgical procedure on the breast (including reconstructive surgery that occurs within 6 weeks of primary resection) or chemotherapy, if patient receives adjuvant chemotherapy, unless wound complications prevent the initiation of treatment; percentage of women having first localization biopsy operation to correctly identify impalpable lesions). There were few investigations of the quality with which this care was delivered. The quality indicators were employed to serve internal quality improvement or external quality oversight.

Database choices reflected study rationale. Small, local databases were used for internal quality improvement, while large databases were used to assess and compare adherence to care across various, larger jurisdictions. The single study linking a quality measurement to outcome noted that reporting the number of affected lymph nodes was linked to both overall and disease-free survival [11].

The only scientifically validated quality measures that were identified assessed QOL (n = 11) and patient satisfaction (n = 1) [12-22]. Of the 12 validated quality measures, 11 were used with reference to treatment and one with regards to diagnosis. None pertained to followup or the documentation of care. Two QOL scales had been specifically validated for use with breast cancer populations. The Functional Assessment of Cancer Therapy Scale (FACT-B, version 3) evaluated the QOL associated with a diagnosis of breast cancer [16]. The European Organization of Research and Treatment of Cancer (EORTC) QLQ-BR23 scale [20] was employed to evaluate the impact of treatment. Other validated instruments included: the Patient Satisfaction Questionnaire [20], Short Form-36 [12,14,16,18,22], EORTC-C30 [14,15]., Medical Outcomes Scale [16,17], Spitzer Quality of Life Index [21], Uniscale [21], Ferrans Quality of Life scale [20], Psychosocial Adjustment to Illness Scale [20], Guttman Health Status Questionnaire [16], and the Linear Analogue Self-Assessment Scale [15].

Overall, where gaps in care appeared to exist, they were generally marked by patterns of under-use rather than lower quality of delivered care. Reports of disparities in breast cancer care amongst groups at risk of being disadvantaged (by age, race, socio-economic status, health insurance) are summarized in Table 2. This includes reports from a wide range of population mixes and sizes, in differing settings, and employing varying standards of optimal care. For example, definitions of "younger" ranged from <40 years to <70 years. Most of the quality indicators were defined in terms of whether or not the indicated care had been received, rather than the quality of the care. No group was advantaged regarding QOL, and the satisfaction study indicated no advantage related to age [17]. Satisfaction was higher among white women and those with government insurance.

Twenty-six quality indicators were identified regarding events surrounding diagnosis, with most not fitting into the project's predefined categories. These measures reflected recommendations that women be seen by specific types of health care professional, for specific reasons, and within certain time frames. The greatest number of studies evaluating a given quality indicator focused on a recommendation pertaining to the use of preoperative diagnosis by fine-needle aspiration cytology, needle biopsy or biopsy (n = 4). Most quality indicators referred to the delivery or receipt of indicated diagnostic care (75%: 18/24). Only five addressed the quality with which specific diagnostic care was delivered. One study observed sound on-study reliability data for an instrument previously validated as a QOL measure [19]. Quality measurements were not found relating to sentinel node biopsy, chest X-ray, bone scan, CT scan, MRI, blood tests, tumor marker status, or genetic testing.

Many more quality indicators were employed to assess treatment (n = 67). The most frequently assessed treatments were adjuvant systemic therapy (n = 25) and radiation therapy (n = 16). The greatest number of studies employing a given treatment-related quality indicator evaluated the appropriate use of breast-conserving surgery (n = 18), and the appropriate use of radiotherapy following breast-conserving surgery (n = 19). Most of the quality indicators referred to the delivery or receipt of indicated treatment (70%: 47/67). Nine quality indicators assessed the quality with which specific treatment care was delivered. Quality measurements were not found relating to reconstructive surgery or neodjuvant systemic therapy, nor to late-stage treatment and palliative care.

Followup care was the focus of five quality indicators, none of which were validated. Specific types of followup care were not predefined.

Of 45 quality indicators relating to reporting/documentation, pathology reporting was the most frequently assessed (n = 42). Reporting the assessment of microscopic margins, and reporting histological type (microscopic) were each evaluated in five studies. Neither surgical nor radiotherapy reporting were the focus of quality measurement.

## Discussion

The measurement of the quality of breast cancer care is in its infancy, despite the fact that breast cancer in women is one of the most-studied areas of healthcare [23].

The clearest observations from this systematic review are that most efforts to measure adherence to quality breast cancer care have centered on whether or not appropriate care was delivered or received (rather than on the quality of this care), focused on treatment, and failed to employ quality indicators formally developed as quality measures. As well, the quality indicators identified did not cover many of the predefined types of diagnostic or treatment care of interest to the funders.

Nearly all quality measurements entailed quality indicators for which no reference was made, or data reported, indicating that they had been developed scientifically as quality measures. Only QOL and satisfaction with care indicators had been validated. Thus, while many measurements were identified, very few were conducted with validated quality measures. In the absence of sufficient data yielded by the application of validated quality measures, the decision was made to forego meta-analysis. For the same reasons, adherence data need to be interpreted with caution. Potential gaps in care compiled in Table 2 unfortunately do not contribute substantially to understanding the divergence of outcomes for American women of different ethnic origins for example.

Malin *et al*. reviewed breast cancer care literature post-1985 [24], and although the present project had a later commencement date, the same quality indicators of breast cancer care were identified.

McGlynn *et al*.'s efforts to establish clinically relevant, valid quality indicators for breast cancer care [8] via a review of the evidence and a peer consensus process, and their findings of under-use, must be considered preliminary. Their study was based on a small number (n = 192) of eligible breast cancer cases; the evidence supporting some (especially treatment) standards was observational by nature, or based on expert opinion; and the quality indicators had not been pilot-tested as measures. Furthermore, patient preference could have been considered [25]. If patient refusal of treatment had been uniformly taken into account, observations of "gaps" may have been different. Under-use of optimal treatment strategies by certain patient groups may arise for a multitude of cultural reasons, and only if measures are sensitive to diverse issues will health care for all citizens be improved. These are some of the issues to be uncovered as part of the formal development of measures.

The most important weakness of the present review is that, due to practical constraints, the "strength" of the clinical evidence base (i.e., the consistency of the results of high quality, appropriately-designed, and adequately-powered primary studies indicating significant links between care and improved outcomes) supporting the definition of each quality indicator (i.e., standard of care) could not be examined. A second limitation is likely that the "level of scientific development" scheme designed especially for this study was itself employed without the benefit of a validation process. Nevertheless, most reports did not describe any validation of their quality indicators, so this limitation did not ultimately affect the results of the review.

Considerable work remains, to define and to measure adherence to standards of breast cancer care. While empirical evidence will likely continue to be collected and synthesized in the pursuit of defining quality breast cancer care, the translation of quality indicators (even with strong support from evidence and clinical consensus) into quality measures with an equally strong psychometric foundation is likely the most pressing need for this field of inquiry to progress. However, before researchers rush headlong into efforts to generate quality measures in the scientific manner described above, it may be wise to appraise the soundness of quality measures under development by ASCO.

ASCO has been developing a set of quality measures relating to stages I-III breast cancer [26]. Their goal is to produce a robust set of largely evidence-based indicators that were being pilot-tested using multiple data sources (e.g., patient survey, ACOS's National Cancer Database) and published with a detailed profile of their reliability (e.g., inter-rater, inter-database), feasibility, and validity. It is hoped that these will be the validated measures required to push forward the field of quality measurement with respect to breast cancer care. It remains to be seen whether or not these quality measures will cover aspects of care (e.g., quality of delivery of care, structural factors) and components of care (e.g., reconstructive surgery, neoadjuvant systemic therapy, sentinel node biopsy, chest X-ray, bone scan, CT scan, MRI, blood tests, tumor marker status, genetic testing, followup, and treatment of recurrent disease and palliative care) identified by the present review as being largely absent from the literature.

Future research efforts to measure adherence to quality breast cancer care could be conducted prospectively, if health care practices and systems were modified to accommodate the required data collection. Virtually all of the efforts to date have involved retrospective data capture. While this strategy reduces the waiting time for collection of especially long-term (e.g., 5 year survival) outcomes, retrospective data collection also makes it difficult to ensure that some of the key factors potentially influencing adherence-to-care data can be observed (e.g., reasons for patient refusal of care).

## Conclusion

A clear, comprehensive understanding of the quality of breast cancer care received by the average citizen is necessary before quality of healthcare may be seriously addressed on a national level [27]. Reliable, validated quality measures with which to identify confidently possible gaps in breast cancer care, and to afford accountability, improvement, and research [28], are the first step to resolving this issue. Some promise is attached to ASCO's ongoing development of breast cancer quality measures, although it will be some time before the results are known. It may be best to proceed with caution before allowing even minor decisions to be guided by any of the adherence data reviewed in this report.

## Competing interests

*Financial competing interest*

Authors of this manuscript (and corresponding review) have not received any reimbursements fees, funding or salary form organizations that may in anyway gain or lose financially from the publication of this manuscript in the past five years prior to start of the corresponding review.

Authors do not hold any stocks or shares in an organization that may in any way gain or lose financially from the publication of this manuscript.

Authors do not hold or are currently applying for any patents relating to the content of the manuscript, nor they have received reimbursements, fees, funding, or salary from an organization that holds or has applied for patents relating to the content of the manuscript.

*Non-financial competing interests*

Authors have no non-financial interests (political, personal, religious, ideological, academic, intellectual, commercial or any other) to declare in relation to this manuscript.

## Authors' contributions

HS: Coordinated the systematic review and lead the conceptual design of the review and manuscript, screened on all levels, verified data, was the primary author of corresponding review, and also drafted the manuscript

VM: assisted with screening and data abstraction, collaborated in conceptualizing the review elements

GL: assisted with screening and data abstraction, collaborated in conceptualizing the review elements

IDG: collaborated in conceptualizing the review elements and provided content expertise

MB: collaborated in conceptualizing the review elements and provided content expertise

MS: specialized search for the corresponding review

AM: technical information specialist assistance

LZ: Information science assistance

PO: system analyst, software (TrialStat), and technical support

CG: coordinated the corresponding systematic review and finalization of manuscript

All authors have red and approved the final manuscript.

## Pre-publication history

The pre-publication history for this paper can be accessed here:



## Figures and Tables

**Table 1:** Quality Indicators Used to Measure Adherence to Standards of Breast Cancer Care

**Type of Quality Indicator**	**n**	Extent of scientific development as a quality measure*
**Diagnosis**		
Appropriate use of imaging, sampling (fine-needle or biopsy) within given time-frame	8	IV
Adequacy of fine-needle biopsy samples	1	IV
Receipt of frozen section of primary operable BC	1	IV
Quality of surgical technique, sampling nodes	2	IV
Quality of hormone receptor assay	1	IV
Quality of life and patient satisfaction relating to diagnosis	2	Iac
Appropriate referral to surgeon	2	IV
Appropriate (timely) attendance at assessment centre, specialist appointment, surgery, receipt of information by patient	5	IV
Efficient diagnosis (few visits to hospital)	1	IV
Appropriate evaluation *vis a vis *guidelines, or at first visit	2	IV
Appropriate specialist knowledge of surgeons	1	IV
**Treatment**		
Appropriate surgical choices – breast conserving, mastectomy, lymph node dissection	7	IV
Timely admission for therapeutic surgery	1	IV
< 3 operations for breast-conserving surgery	1	IV
Evidence of discussion of surgical options	1	IV
Appropriate use, timeliness of initial radiotherapy	6	IV
Quality of radiotherapy planning, fractionation, radiation field distribution	7	IV
Regional recurrence	1	IV
Appropriate use of radiotherapy for regional recurrence, palliation	1	IV
Appropriate use (or not) of adjuvant systemic therapy	23	IV
Chemotherapy quality of administration – dosages and availability of procedure manual	2	IV
Quality of life, satisfaction with treatment	6	Ia, Iac
Participation in decision-making, receipt of sufficient information re. treatment	2	IV
Qualifications of doctors	2	IV
Appropriate referrals to specialists	2	IV
Appropriate treatment choices, sequences	5	IV
**Followup**		
Appropriate followup mammography, use of guidelines	2	IV
Recurrence within 5 years	2	IV
Appropriate use of prophylactic radiotherapy in women with high risk of flap recurrence	1	IV
**Reporting/Documentation**		
Pathology reporting/documentation	42	IV
Imaging reporting/documentation – size of mammographic abnormality	1	IV
Chemotherapy reporting/documentation	2	IV

n = number of different quality indicators regarding this type; *extent of scientific development of quality indicator: Level Ia = pre-study data indicating consistently sound psychometric properties; Iac = pre- *and *on-study data indicating consistently sound psychometric properties; IV = no pre- *or *on-study psychometric data

**Table 2:** Quality of Treatment of Breast Cancer in Women of Different Age, Socio-economic Status, Race or Ethnicity

**Number of reports indicating the group is advantaged, according to measure**																	
**Measure**	**Age**			**Race**			***Health Insurance***			***Education***			**Income**			**Urban**	**Rural**
			
	**Young**	**Old**	**All**	**Colored**	**White**	**All**	**Private**	**Government**	**All**	**High**	**Low**	**All**	**High**	**Low**	**All**		

Pre-operative mammogram	1					1			1								
Referral to surgeon by G.P.		1															
Diagnostic evaluation *per *guidelines		1					1										
Satisfaction with care			1		1			1									
Appropriate breast-conserving surgery and axillary lymph node dissection	7						3		2	1		2	1			1	
Appropriate radiotherapy	5						2										
Appropriate systemic therapy	5		3		2	1	1			1			1				
Appropriate treatment alternatives	3									1			1				
Use/Quality of Radiotherapy			1		3	2			1								
Improvement in QOL over time			1														
Appropriate axillary lymph node dissection and chemotherapy				1													

## References

[B1] Cancer Facts and Figures 2004. http://www.cancer.org/downloads/STT/CAFF_finalPWSecured.pdf.

[B2] Cancer Facts & Figures 2003. http://www.cancer.org/downloads/STT/CAFF2003PWSecured.pdf.

[B3] Breast Cancer Facts & Figures 2003–2004. http://www.cancer.org/downloads/STT/CAFF2003BrFPWSecured.pdf.

[B4] Hussey PS, Anderson GF, Osborn R, Feek C, McLaughlin V, Millar J, Epstein A (2004). How does the quality of care compare in five countries?. Health Aff (Millwood).

[B5] Institute of Medicine Committee on the National Quality Report on Health Care Delivery (2001). Envisioning the National Health Care Quality Report.

[B6] Institute of Medicine (2000). Crossing the Quality Chasm: A New Health System for the 21st Century.

[B7] McGlynn EA, Asch SM, Adams J, Keesey J, Hicks J, DeCristofaro A, Kerr EA (2003). The quality of health care delivered to adults in the United States.[comment]. N Engl J Med.

[B8] Chassin MR, Galvin RW (1998). The urgent need to improve health care quality. Institute of Medicine National Roundtable on Health Care Quality. JAMA.

[B9] Measuring the quality of breast cancer care inwomen 2004. http://www.ahrq.gov/downloads/pub/evidence/pdf/brcancer/brcancare.pdf.

[B10] Moher D, Cook DJ, Eastwood S, Olkin I, Rennie D, Stroup DF (1999). Improving the quality of reports of meta-analyses of randomised controlled trials: the QUOROM statement. Quality of Reporting of Meta-analyses. Lancet.

[B11] Ottevanger PB, De Mulder PH, Grol RP, Van Lier H, Beex LV (2002). Effects of quality of treatment on prognosis in primary breast cancer patients treated in daily practice. Anticancer Res.

[B12] Jansen SJ, Stiggelbout AM, Nooij MA, Noordijk EM, Kievit J (2000). Response shift in quality of life measurement in early-stage breast cancer patients undergoing radiotherapy. Qual Life Res.

[B13] Osoba D, Burchmore M (1999). Health-related quality of life in women with metastatic breast cancer treated with trastuzumab (Herceptin). Semin Oncol.

[B14] Chie WC, Huang CS, Chen JH, Chang KJ (1999). Measurement of the quality of life during different clinical phases of breast cancer. Journal of the Formosan Medical Association.

[B15] Bernhard J, Hurny C, Coates AS, Peterson HF, Castiglione-Gertsch M, Gelber RD, Goldhirsch A, Senn HJ, Rudenstam CM (1997). Quality of life assessment in patients receiving adjuvant therapy for breast cancer: the IBCSG approach. The International Breast Cancer Study Group.[erratum appears in Ann Oncol 1998 Feb;9(2):231]. Ann Oncol.

[B16] Frazer GH, Brown CH, Graves TK (1998). A longitudinal outcome assessment of quality of life indicators among selected cancer patients. Journal of Rehabilitation Outcomes Measurement.

[B17] Molenaar S, Sprangers MA, Rutgers EJ, Luiten EJ, Mulder J, Bossuyt PM, van Everdingen JJ, Oosterveld P, de Haes HC (2001). Decision support for patients with early-stage breast cancer: effects of an interactive breast cancer CDROM on treatment decision, satisfaction, and quality of life. J Clin Oncol.

[B18] Bower JE, Ganz PA, Desmond KA, Rowland JH, Meyerowitz BE, Belin TR (2000). Fatigue in breast cancer survivors: occurrence, correlates, and impact on quality of life. J Clin Oncol.

[B19] Northouse LL, Caffey M, Deichelbohrer L, Schmidt L, Guziatek-Trojniak L, West S, Kershaw T, Mood D (1999). The quality of life of African American women with breast cancer. Research in Nursing & Health.

[B20] Dow KH, Lafferty P (2000). Quality of life, survivorship, and psychosocial adjustment of young women with breast cancer after breast-conserving surgery and radiation therapy. Oncol Nurs Forum.

[B21] Perez DJ, Williams SM, Christensen EA, McGee RO, Campbell AV (2001). A longitudinal study of health related quality of life and utility measures in patients with advanced breast cancer. Qual Life Res.

[B22] Mor V, Malin M, Allen S (1994). Age differences in the psychosocial problems encountered by breast cancer patients. J Natl Cancer Inst Monogr.

[B23] Malin JL, Asch SM, Kerr EA, McGlynn EA (2000). Evaluating the quality of cancer care: development of cancer quality indicators for a global quality assessment tool. Cancer.

[B24] Malin JL, Schuster MA, Kahn KA, Brook RH (2002). Quality of breast cancer care: what do we know?. [Review] [87 refs]. J Clin Oncol.

[B25] McGlynn EA (2003). Selecting common measures of quality and system performance. Med Care.

[B26] Schneider EC, Epstein AM, Malin JL, Kahn KL, Emanuel EJ (2004). Developing a system to assess the quality of cancer care: ASCO's national initiative on cancer care quality. J Clin Oncol.

[B27] McGlynn EA, Brook RH (2001). Keeping quality on the policy agenda. Health Aff (Millwood).

[B28] Galvin RS, McGlynn EA (2003). Using performance measurement to drive improvement: a road map for change. Med Care.

